# Nervous Dyspepsia (Neurasthenia Gastrica)—Its Diagnosis and Treatment, with Report of Cases*Read by invitation before the East Texas Medico-Chirurgical Society at Crockett, Texas, December 1, 1904.

**Published:** 1906-01

**Authors:** John T. Moore

**Affiliations:** Galveston, Texas


### TEXAS MEDICAL JOURNAL.

ESTABLISHED JULY, 1885.

PUBLISHED MONTHLY.—SUBSCRIPTION $1.00 A YEAR.

VOL. XXI. AUSTIN, JANUARY, 1906. No. 7.

The pnblisher is not responsible for the views of contributors.

For Texas Medical Journal.

### Nervous Dyspepsia (Neurasthenia Gastrica)—Its Diagnosis and Treatment, With Report of Cases.*

BY JOHN T. MOORE, M. D., GALVESTON, TEXAS.

The topic I have decided upon for consideration tonight is one that I feel sure is of more or less interest to every one of us who does a general or consulting practice. I have nothing new to offer you, but wish to present several cases and discuss briefly the diagnosis and treatment of a neurosis that is quite common in this country.

Leube, of Germany, originated the term nervous dyspepsia to designate a condition in which there is a train of symptoms of a markedly nervous character with apparent disease of the stomach and intestines, there being many symptoms of indigestion present, yet no organic lesion is found to exist in the stomach or bowel.

Einhorn ("Diseases of the Stomach," 5th edition) says: "Nervous dyspepsia may best be characterized by the existence of manifold clinical symptoms without any organic lesions whatever."

Many clinicians have objected to the use of the term as too indefinite and general, but an examination of the literature will convince one that the terms are generally used and understood by writers upon digestive disorders.

Riegel ("Diseases of the Stomach—Nothnagel's System") makes the following classification of neuroses of the stomach: (1)

*Read by invitation before the East Texas Medico-Chirurgical Society at Crockett, Texas, December 1, 1904.

motor neuroses, (2) secretory neuroses, (3) sensory neuroses. William Osler, of Johns Hopkins Hospital, follows this classification in his discussion of the disorder.

Progress has been very great during the past thirty years, and more especially in the last ten years, in differentiating the various diseases of the stomach, so that now by the use of the stomach-tube, chemical examinations, and clinical methods, one is able to diagnose and definitely classify many of the disorders of the stomach. These exact methods of diagnosis are taken up rather slowly by the great army of physicians throughout the country, though the adoption of the best methods in the examination and diagnosis of diseases of the stomach would render this line of practice much more interesting and satisfactory. One is able to get results that compare very favorably with the treatment of other disorders, and oftentimes brilliant results may be achieved.

*Etiology.*—Nervous dyspepsia is a mixed neurosis, and many times follows some debilitating disease or comes on after a continuous disregard of the laws of correct living with reference to temperance, to work, eating, drinking, and sleeping. Constant application to business under circumstances of worry and apprehension is a frequent cause of this disorder.

Oversexual indulgence, coitus reservatus, and abdominal conditions of the genito-urinary organ are frequent causes.

Uterine disorders and displaced (over-movable) kidneys are frequent causes. Cardiac and renal disorders should always be considered as causes that produce nervous dyspepsias. The various blood dyscrasia as chlorosis, malaria, and intestinal parasites, are frequent causes.

The disease is said to be more frequent among women, though my cases have shown no difference in the sexes.

The affection is more common in the young and middle-aged. Einhorn says that the disease is more frequent from thirty to forty-five years.

Tobacco, alcohol, and coffee should be especially mentioned, as they are frequently causes.

*Symptoms.*—Leube speaks of the irritation of the nervous system in healthy individuals caused by the ingestion and digestion of foods, as evidenced by the confused conditions of the head, disposition to sleep, fatigue, and a sense of fullness and pressure after meals. He says further that if to these be added a poor appetite, eructations, heart-burn, nausea, headache, vertigo, etc., yet digestion is accomplished in a normal time, nervous dyspepsia may be suspected. He advises the use of a stomach-tube in all cases to ascertain whether the stomach empties itself within the normal limit, say in seven hours after taking the ordinary meal.

There is nothing characteristic of the stomach after a test meal, the acidity being increased in some, normal or diminished in others. The examination of the contents are of most value in excluding other affections of the stomach. In most cases there is tenderness over the epigastrium and there may be tender points between the shoulders behind.

There is frequently a marked distension of stomach and bowels, causing belching or passage of gas from the bowels. Many complain of pain in the region of the heart. One kind of food agrees about as well as another. The stomach empties itself in the normal time, the washings rarely ever bringing anything away after six or seven hours from time of taking food.

There is rarely any vomiting, though there is often a feeling of nausea or emptiness about the stomach. Patients are apt to dwell upon their symptoms, and to magnify ills in various ways. Some are melancholy, being disposed to mope around or sleep a great deal. Irritability of temper is quite common.

In the differential diagnosis, one should consider and exclude chronic catarrh of the stomach, ulcer, and cancer.

The absence of evidence of these; the normal digestion, except to cause distress, of all articles of food, and the presence of a line of nervous symptoms associated with the complaint of indigestion, will make out the case.

Of course, one has to consider the various etiological factors of this disease in making a diagnosis, as, for example, a great press of business duties, anxiety, etc. The following cases will probably illustrate some of the different phases of the disease:

*Case No. 1.* Age 30 years; married; occupation, bookkeeper; family history good. Smokes cigarettes to excess, and is a hearty eater. Dissipates a good deal by going out with his wife, who is fond of society. He has done this for several years. Has been married four years. He has long hours at business, rarely getting to sleep more than six or seven hours per day. Sleeps poorly, staying awake much of the time he is in bed, worrying over his business. Has irregular meals.

About three months before consulting me, the patient states that the indigestion, which has troubled him more or less for four or five years, began to get worse. Felt a fullness about the stomach after eating. Had much belching of gas after meals. Pain and discomfort over stomach with spells of dizziness or vertigo. Suffered from pain in the region of his heart. Has heavy drowsy feelings at times. Good appetite most of the time. Bowels constipated most of the time. He carries a bottle of aromatic spirits of ammonia with him for the spells.

A careful examination was made, and the heart and lungs found to be normal. No evidence of tumor about the abdomen. Some tenderness present over the abdomen, but no distension of the stomach. Some slight peristaltic waves could be seen over the abdomen. Reflexes normal except the patellar slightly increased. Marked tache cerebrale. A test-meal showed free hydrochloric acid 20 per cent. The stomach emptied itself in less than seven hours. Blood examination showed an anemia, though not marked. Urinary examination revealed nothing abnormal except a large amount of indican.

*Note.* Upon being more closely questioned, the patient stated that during his married life he has been practicing coitus reservatus to prevent having children, and that since this practice he has been growing worse; and, in fact, dates his trouble from about that time.

*Case No. 2.* Mrs. H., age 30 years; married twelve years, and has five children, the eldest being 11 and the youngest 2 years of age. She had typhoid fever when 16 years of age, otherwise has been healthy. Regular in menstruation. She was operated upon for laceration of the cervix seven years ago, and was curetted three years ago. She now complains of being quite nervous, and occasionally has a "spell" which one would probably call hysterical. She has nausea, fullness of the stomach after eating, much belching of gas one-half hour after eating; appetite poor and very capricious; wants sauces, pickles, etc. Has been suffering in this way about two months.

Examination of heart, lungs, and nervous system showed nothing abdominal. Urine normal; stomach normal in size and slightly tender. Vaginal examination revealed a leucorrhea and a laceration of the left side of the cervix.

A test-meal was given and showed hydrochloric acid 20 per cent, and a combined acidity of 45. Starch digestion complete, and the stomach free of food in less than seven hours.

*Case No. 3.* Mr. K., age 31; married; one child; occupation, merchant; family history excellent; previous health good, except typhoid fever in 1892. Patient states that he has been closely confined to his business, working all day and part of the night.

Temperate in his habits of eating and drinking. He noticed about four months ago that he began to get weak. Has lost about twenty-five pounds in weight; no cause; easily fatigued; very nervous. Vomits after eating, especially if he eats heartily; fullness about the stomach; belches gas.

Examination revealed nothing abnormal about the heart, lungs, kidneys, liver, etc. Stomach a little distended; abdomen normal. An Ewald-Boas test-meal was given, and the contents showed no free hydrochloric acid; combined acidity 42; starch digestion incomplete.

*Case No. 4.* Mr. L., age 22; married one and a half years; one child; family history not important; occupation, delivers laundry. Drinks beer only occasionally and moderately; habits regular. Says that several months now has been suffering from pain over the stomach and around the heart. The pain is relieved by eating, but returns in about two hours. Much eructation of gas; bowels constipated; is easily fatigued; sleeps poorly and is very nervous; little things cause much irritation.

Nothing abnormal was found about the heart, lungs, kidneys, or other organs. Stomach examination of test-meal showed free hydrochloric acid 60, combined acidity 78. Starch digestion complete. Washed out the stomach after a regular dinner, and found the stomach empty.

*Case No. 5.* C. M., age 46; married; two children; occupation, merchant; family history good; smokes tobacco; sexual habits temperate; moderate eater; closely confined to business for two years; worried about business during this time; business is now prospering, and there is no cause for worry. Complains of constipation, indigestion, and general nervousness. Belches much gas and passes a great deal from the bowels. Has lost about twenty pounds in weight, is easily fatigued, and at times feels very much depressed. Has little sexual desire.

Examination showed heart, lungs, kidneys, liver, etc., to be normal; pulse 88; tache cerebrale very marked. Test-meal showed free hydrochloric acid 18, combined acidity 52, and starch digestion incomplete. Twelve days later a test-meal showed hydrochloric acid 10, combined acidity 38, and starch digestion complete.

Patient stated that he has a strong suspicion of people where he had no grounds for the same. He recognized that before his trouble began there was no suspicion and no occasion whatever for it.

*Treatment.* The treatment will vary very much in each case. One must hunt out the underlying cause and remove it if possible. A careful study of the stomach contents should be made so as to arrive at a correct idea of the stomach secretions and functions. In the majority of these cases the stomach symptoms are secondary to a general neurosis, hence all plans of treatment must be directed to the rebuilding of the nervous system.

Residence at the seashore, combined with bathing, fishing, boating, and other out of door recreations are most useful measures where the patient is not too weak. Many of the cases, however, require complete rest in bed for several weeks before beginning this line of treatment. It is my practice to put the patient at rest in bed under close observation until I get the case thoroughly in hand. Sleep and rest are necessary before improvement can begin. The wet pack will do more to produce sound, restful sleep than many of the sleep-producing drugs. Occasionally it may be necessary to give chloral combined with the bromid of soda. Trional is valuable. I never use the opium preparations. One must invariably be removed from all irritating influences and cares of business, or of the household.

A morning bath in cold water and a vigorous rub with a coarse towel is of much benefit. Some cases do better under the influence of the cold sheet, and then vigorous rubbing. Many of these cases have been placed upon a starvation diet, thinking they could not digest a general diet. It is my experience that most of these cases can digest pretty nearly anything. They certainly do so after a little treatment. A highly nutritious diet is necessary and I have them fed well.

Stomach lavage six or seven hours after meals twice or three times a week helps a great deal. I wash out the stomach after the evening meal. Massage and electricity are excellent measures. The bowels must be looked after carefully, as quite a few of these patients suffer from constipation. Tonics are of great service, especially nux vomica, condurango, and the glycerophosphates. Iron and arsenic are good to give in cases of anemia.

After there is improvement sufficient to allow patient to go out sea-bathing about three times a week, preferably early in the morning before breakfast, is of great benefit. Fishing should be encouraged,—it occupies the mind sufficiently to prevent self-examination. Encouraging words from the doctor is one of the most helpful measures. Take occasion every now and then to assure the patient that he is going to get well, and show wherever you can that he has improved.

*Literature.* J. C. Hemmeter, 2d edition, "Diseases of the Stomach"; C. A. Ewalt, "Diseases of the Stomach"; Van Valzah and Nisbet, "Diseases of the Stomach"; F. Reigel (Nothnagel's Practice) "Diseases of the Stomach"; Max Einhorn, "Diseases of the Stomach."

